# A Lecture on the Principles and Practice of Dental Surgery

**Published:** 1859-01

**Authors:** Alfred Carpenter


### SELECTED ARTICLES.

### ARTICLE XIV.

*A Lecture on the Principles and Practice of Dental Surgery.*
By Alfred Carpenter, M. B., M. R. C. S., &c.

(Continued from October Number.)

The next subject that will engage our attention will be that of *tumors*, a term often applied to any swelling, but most properly restricted to a morbid growth, either by the enlargement of natural parts in an unnatural manner, or by the organization of matter which is unnatural, and not properly found in the tissues of the human body.

Tumors may be divided into two classes, those which from their intractibility and dangerous character may be called *malignant*, and those which are not in themselves attended with any danger, and being comparatively innoxious may be called *benign*.

Malignant tumors are those enlargements of parts depending upon some constitutional vice, some unnatural state of the system, by which they are developed, take root, and grow to a considerable size, frequently in a short time, but occasionally in a very slow manner; they resist all remedies, and tend to convert the surrounding tissues into their own substance. They spread rapidly, ulcerate in a most virulent manner, destroy muscles, bones, bloodvessels, producing frequent and exhausting hemorrhages, and at times are exceedingly painful, as well as very frequently fatal.

I can of course scarcely do more than enumerate their names, and you must, if you please, search out for yourselves the way to distinguish them, as well as some part of the treatment to be recommended for their removal. They may have their origin in the bony substance of the jaws, or the tissue of the gums, and, in fact, in any part of the system; and it will be highly important for you to make out their origin and attachments, before deciding what steps should be taken for their removal; but, however, unless you are intending to become members of the college of surgeons, you will scarcely expect to have to perform any of these operations.

The principal malignant tumors or growths, are—

Hard carcinoma or cancer, or scirrhus;
Mallanosis;
Medullary sarcoma;
Osteo sarcoma.

The *Hard Cancer, or Scirrhus*, is known by the character of the pain, darting, lancinating; by its excessive hardness with softer points—by its tendency to involve the skin and all the surrounding tissues. In tumors that are non-malignant, you may generally move the skin freely over the swelling, but in cancer this cannot be done. You will also find, that there is a so-called cancerous cachexia, that is, a peculiar appearance of the patient, which, once observed in a bad case of cancer, is never forgotten.

*Mallanosis* is much less frequently seen as a disease of bone; it consists in a morbid product, presenting a black or deep brown color, unorganized, and differing in the form it assumes; and also in its consistence it is frequently mixed up with scirrhus or encephaloid disease, and is evidently a branch of the same tree.

*Medullary Sarcoma* is another tribe of the same character, in appearance much like the substance of the brain, hence another name, encephaloid; it frequently arises in the cancelli of bone; it may have its origin in the antrum; it partly absorbs, and partly forces open the shell of the bone, and commits most awful ravages in a very short space of time.

*Osteo Sarcoma* is a kind of cartilaginous growth, consisting of numerous cysts—containing reddish fluid, and having plates and spiculæ of bone dispersed throughout.

There are some other divisions, as *Cystic Sarcoma* and *Colloid.* Now, all these diseases return again if extirpated; they have a constitutional cause, although, in the first instance, they may have had a local origin. I know of no means of curing them.

Not so the non-malignant tumors, they are are all capable of cure, or relief to some extent; they may have their origin in the gum, or in the mucus membrane adjacent to it, or in the periosteum of the bone, or of the teeth, or in the jaw bones, or the teeth themselves. They are—

1. Epulis
2. Epithelial cancer
3. Hypertrophy
4. Fibrous growths
5. Fatty growths
6. Membraneous cysts
7. Erectile tumors
8. Osseous tumors.

*Epulis* is a form of disease originating in the gum, as a simple enlargement, or hypertrophy, or as a circumscribed tumor growing from the gum, and occasionally attached to it by a narrow pedicle; it is usually slow in its progress, and forms a hard painless swelling, projecting from the alveolar border of the jaw, at first smooth, it afterwards becomes rough and tubercular, softens, and ulcerates, then bleeds freely. As the swelling increases, it extends around the teeth, and encloses them, and often seems to have originated from their sockets; sometimes it is merely indurated gum, and in other cases it has dense fibrous tissue, with irregular scattered particles of bone in its substance. It is easily reproduced, and will certainly reappear unless all portions of it have been removed.

*Epithelial Cancer* originates also in the gum, or the adjacent mucous membrane; it begins with swelling and induration; it then becomes wartlike, and rapidly ulcerates; it spreads slowly from its first habitat to all parts and all structures of the mouth, it is not at first painful, and it is not long before it effects the general health; it is undoubtedly malignant in its character, but there is hope for the patient if it is entirely and early removed from the mouth. It is therefore highly important that you should recognize this disease, as well as epulis, before it has crept irregularly and indefinitely into the surrounding structures.

*Hypertrophy* is a term of general application, when the tissue of any part is thickened, without containing anything more abnormal than the deposit of coagulable lymph, or fibrous tissue; thus the gums may be hypertrophied, the periosteum may be thickened, or the bones themselves enlarged; you will do well to study the meaning of the term, as applied to general pathology; you will then be able to understand its application to diseases of the jaw.

*Fibrous Growths*, or tumors, consist of nodules, or lumps of fibrous tissue, variously developed, as regards situation; the tumors may be white, compact and opaque, with particles of bone scattered through it, or gray and dense fibrous tissue, originating either in the interior of the lower jaw bone, or from its alveolar border and outer surface, or it may be almost cartilaginous in its character.

Mr. Stanley states, that there is an instance of this growth in the museum of the college of surgeons, of great magnitude, taken from the jaw of an adult female.

Sometimes we meet with *fatty growths*, swellings, containing granules of fat intermingled with cells filled with glairy fluid, and generally originating in the interior of the jaw.

*Membraneous Cysts*, again, contain a glairy fluid, originating within the jaw. Mr. Stanley, our great author upon diseases of the bones, states that "these cysts in enlarging usually cause expansion of the walls of the jaw, and they are found to possess, more or less, complete osseous parietes, apparently formed by hypertrophy of the cancellous texture of the jaw; occasionally the membraneous cysts, instead of expanding the walls of the jaw, cause the absorption of its outer wall, so that the tumor they form projects on the outer side of the jaw. This disease is usually of slow growth."

Another form of swelling consists of *erectile tissue,* also originating in the interior of the jaw; this tissue being of a character allied to the aneurismal tumors, which are also found in this part of the body, as well as everywhere else. An aneurismal tumor may generally be detected by the throbbing or pulsating sensation which it communicates to the hand upon pressure. You must be especially careful to detect these, should they ever come to your notice, as highly dangerous, and even fatal results might ensue if you thoughtlessly treated them as you would into a mere gum boil.

The last kind of tumor I shall consider is perhaps the most important.

*Osseous Tumors or Exostosis.*—Some of these are hard, others are not so hard, being composed of cancellous texture, or they may be partly osseous and partly fibrous. The true exostosis is known by being hard, free from pain, and slow in its growth; there is no constitutional mischief, and the surrounding parts are healthy. Its microscopic as well as chemical qualities, are precisely the same as true bone, at least the differences in the proportions of the ingredients may be referred to the different degrees of hardness in the specimens analyzed, rather than to actual difference in composition. Most exostoses have cartilage for their base, and a cartilaginous growth preceded the formation of bone in the first instance, the osseous substance being deposited in the centre of the cartilage, and it increases like the ossification of bone in a young subject. Exostoses vary much in figure, some being attached to the bone by a narrow neck, and others by a broad and lengthened base. Some are movable by reason of the very narrow neck with a cartilaginous structure, or from the bony neck having been broken. Their surface may be smooth or nodulated, and they increase in an irregular manner, sometimes rapidly, and then again are quite stationary. They at times seem hereditary, and I am acquainted with a family in which every one of the children have exostoses on some part of their skeleton. Their correct diagnosis is important; recollect then, the hardness, the freedom from pain, the absence of constitutional disturbance, and of surrounding disease; the evident connection with the bone from which it springs, and its slow growth, and you will not have any difficulty in deciding its nature. When the tumor is more of a cartilaginous than of a bony nature it is usually called an enchondroma.

The situation of an exostosis is always an important consideration; the tumor is in itself harmless, but when from situation it presses upon nerves, or repels or impedes movement, it is necessary to remove it, and its removal must be complete and entire, or it will return again. Many other plans have been recommended for its cure, but the knife is by far the most effectual, and the only certain cure. The most frequent seat of exostoses which will fall under your notice, will be the fangs of the teeth; they are occasionally met with upon the lower jaw bone, but very seldom upon the upper. Enchondromatous tumors occur, occasionally upon the alveolar processes of the upper jaw, but you will seldom meet with them.

Time warns me to bring my discourse to a conclusion. There are of course great numbers of diseases passed by in silence, for I can only look upon this course as preliminary to a more extended series at a future period. I thank you heartily for your kind attention, and would beg you to recollect, that merely attending a lecture will never teach you how to practice your profession; you must study for yourself, and read carefully upon every subject that will be likely to fall under your notice. Study it from its foundation, consider it in its every phase, and you will then be in a better position to advance the practical part of dentistry when you are your own masters.

As regards the works that will be useful to you, you ought to pay particular attention to your own dental authors, among whom stands pre-eminently forward your highly esteemed president, whose work will long be the students manual. His presence prevents me saying all that I consider due to it. You will, however, find more dental authorities among the Americans, than in our own country. I believe Dr. Harris is the most esteemed, whilst I have derived great advantage from Dr. Bond's observations. As regards general surgery and pathology, you will find Hunter's works worth your attention. Simon's Lectures upon Pathology, Dr. Carpenter's Physiology, and the first lectures of Dr. Watson upon Medicine; Stanly upon the Bones—Travers on Constitutional Irritation, will all be worth serious study, and you will find that many of the points I have advanced are upon their authority.

There is one point I will also take the liberty of recommending to the council of your society—never rest for one moment from your labors until you have established a dental hospital and dispensary. The poor of this great metropolis require aid, and your students require ocular instruction. Your course of study will never be complete without clinical lectures, and the positive education afforded by hospital practice: do not lose sight of this point, as you value the advancement of your profession.

Gentlemen, I thank you for your kind and attentive hearing. [*Lon. Quar. Jour. Dent. Sci.*

